# A Novel Pathway Links Oxidative Stress to Loss of Insulin Growth Factor-2 (*IGF2*) Imprinting through NF-κB Activation

**DOI:** 10.1371/journal.pone.0088052

**Published:** 2014-02-18

**Authors:** Bing Yang, Jennifer Wagner, Nathan Damaschke, Tianyu Yao, Shelly M. Wuerzberger-Davis, Moon-Hee Lee, John Svaren, Shigeki Miyamoto, David F. Jarrard

**Affiliations:** 1 Department of Urology, University of Wisconsin School of Medicine and Public Health, Madison, Wisconsin, United States of America; 2 Department of Oncology, McArdle Laboratory for Cancer Research, University of Wisconsin, Madison, Wisconsin, United States of America; 3 Comparative Biosciences, University of Wisconsin, Madison, Wisconsin, United States of America; 4 University of Wisconsin Carbone Comprehensive Cancer Center, Madison, Wisconsin, United States of America; 5 Environmental and Molecular Toxicology, University of Wisconsin, Madison, Wisconsin, United States of America; UC Davis Comprehensive Cancer Center, United States of America

## Abstract

Genomic imprinting is the allele-specific expression of a gene based on parental origin. Loss of imprinting(LOI) of Insulin-like Growth Factor 2 (*IGF2*) during aging is important in tumorigenesis, yet the regulatory mechanisms driving this event are largely unknown. In this study oxidative stress, measured by increased NF-κB activity, induces LOI in both cancerous and noncancerous human prostate cells. Decreased expression of the enhancer-blocking element CCCTC-binding factor(CTCF) results in reduced binding of CTCF to the H19-ICR (imprint control region), a major factor in the allelic silencing of *IGF2*. This ICR then develops increased DNA methylation. Assays identify a recruitment of the canonical pathway proteins NF-κB p65 and p50 to the CTCF promoter associated with the co-repressor HDAC1 explaining gene repression. An IκBα super-repressor blocks oxidative stress-induced activation of NF-κB and *IGF2* imprinting is maintained. *In vivo* experiments using IκBα mutant mice with continuous NF-κB activation demonstrate increased *IGF2* LOI further confirming a central role for canonical NF-κB signaling. We conclude CTCF plays a central role in mediating the effects of NF-κB activation that result in altered imprinting both *in vitro* and *in vivo*. This novel finding connects inflammation found in aging prostate tissues with the altered epigenetic landscape.

## Introduction

Imprinting is the allele-specific expression of a gene based on its parent of origin, and it plays an important role in normal development. Imprinted genes appear remarkably sensitive to environmental changes including diet and oxidative stress. Imprinting is disrupted in blastocysts cultured in high-oxygen environments [1] and is altered *in vivo* by excess maternal folate [2]. Recently, interest has arisen regarding disruption of imprinting and other epigenetic features during aging that may alter gene expression and lead to disease. Whether inflammation, a common feature in aging-related cancers such as prostate and colon [3], [4], may alter imprinting patterns is unknown.

Age-associated loss of imprinting (LOI) of the Insulin-like growth factor 2 (*IGF2*) and other genes has been demonstrated in mouse and human tissues [5], [6]. In the prostate, decreased expression of the enhancer-blocking element CCCTC-binding factor (CTCF) leads to reduced binding to the *IGF2*-H19 imprint control region and loss of imprinting (LOI) in older animals [6]. CTCF is a zinc finger protein that functions as an insulator to block enhancer access to the silenced *IGF2* promoter [7]. It also acts to protect regions of the genome from DNA methylation [8]. Notably, LOI at *IGF2* is more extensive in men with associated cancer supporting a role in cancer promotion with aging [6].

A shift in the prooxidant-antioxidant balance results in excess reactive oxygen species (ROS) during aging. This is manifest, in part, by an increase in the frequency of inflammation and histologic lesions in aging tissues such as prostatic post-inflammatory atrophy (PIA). An accumulation of the oxidative adducts 8-hydroxy-2′-deoxyguanosine (8-OHdG) also occurs in aging prostate tissues [9]. NF-κB plays a pivotal role in regulating the cellular stress response to oxidative injury and infection. NF-κB levels increase in the prostate and other aging tissues [10]–[12]. In unstimulated cells, NF-κB family proteins exist as heterodimers or homodimers that are sequestered in the cytoplasm by virtue of their association with a member of the IκB family of inhibitory proteins. Canonical activation results in degradation of IκB, and NF-κB translocation into the nucleus where it binds to specific response elements and recruits additional cofactors. Blocking NF-κB activation in the skin of aged mice reverts the global gene expression program and tissue characteristics to those of young mice [11].

The mechanism underlying the association between increased oxidative stress and altered imprinting is unknown. CTCF is altered with cellular stress [13]. In the present study we find oxidative stress induces NF-κB binding to the CTCF promoter leading to decreased expression. This, in turn, results in a loss of CTCF binding to the ICR and bialleleic *IGF2* expression. Mutation of IκBα resulting in the overactivation of canonical NF-κB directly results in CTCF loss in the mouse and specifically confirms the role of this pathway in altered imprinting. These data demonstrate a novel link between oxidative stress and loss of imprinting and suggest that modulating NF-κB may prevent age-related alterations in the epigenome.

## Results

### Induction of NF-κB by oxidative stress is associated with *IGF2* loss of imprinting (LOI) in viable prostate cells

**Figure 1:**
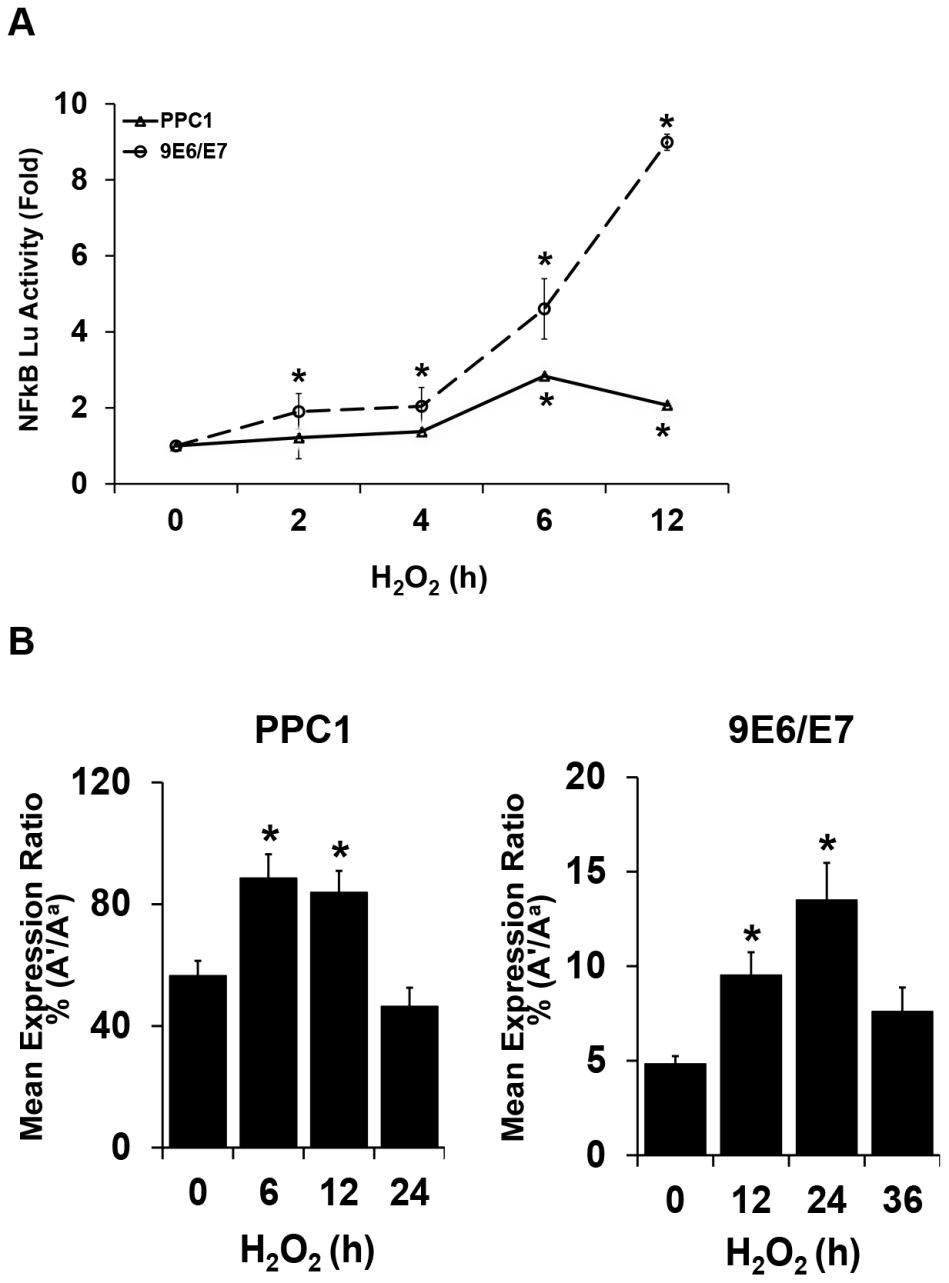
Oxidative stress results in NFkB activation and *IGF2* loss of imprinting. (A) Oxidative stress induces NF-κB activation in prostate cells. PPC1 or 9E6/E7 NF-κB reporter stable cells were treated with 800 µM or 1600 µM of H_2_O_2_, respectively, for the indicated times. NF-κB luciferase (Lu) activity was plotted against no treatment controls ( = 1), and the activity change in the NF-κB reporter is shown as the fold change. Values are expressed as mean+/−S.D. of three independent experiments measured in duplicate. * *P*<0.05, (t-test). (B) Oxidative stress induces loss of *IGF2* imprinting in prostate cells. FluPE was performed as reported to assess individual allelic RNA expression for *IGF2* [6]. Values are expressed as mean+/−S.D. of three independent experiments. *, *P*<0.05 (t-test).

*IGF2* LOI is an aging-related epigenetic event [6] that we postulate may be modulated by inflammation, a common feature associated with prostate and colon cancer development [3], [4]. Inflammation induces NF-κB, a stress-induced transcription factor with a pivotal role in regulating cellular signaling [14], [15]. We engineered stable NF-κB reporter PPC1 cancer and 9E6/E7 epithelial prostate cell lines harboring the firefly luciferase reporter gene under the control of a minimal (m)CMV promoter and tandem repeats of the NF-κB transcriptional response element to measure NF-κB transcriptional activity. Prostate cancer and normal epithelial immortalized cell lines were chosen, in part, because they were informative for imprinting quantitation. 9E6/E7 has a longer doubling time and is nontumorigenic, in contrast to the cancer line PPC1 [16]. Dose response studies were performed to maximize the detection of NF-κB activity and viability while minimizing apoptosis induction (<2% at 6 hours, data not shown). After being normalized to the control reporter, NF-κB activity peaked in PPC1 (2.8 fold) at 6 hr and in 9E6/E7 (9.5 fold) at 12 hr when exposed to 800 µM and 1600 µM of H_2_O_2_, respectively (Fig. 1A).

The allele-specific expression of *IGF2* in the two cell lines after exposure to H_2_O_2_ was determined. Both cell lines contain a polymorphism (G/C) in exon 7 within the *IGF2* coding sequence allowing allele-specific expression to be quantitatively assessed using FluPE as previously described [6]. A relaxation of *IGF2* imprinting developed in both cell lines after H_2_O_2_ treatment in a time-dependent manner (Fig. 1B). The reexpression of the silenced allele was divided by the expressed allele in these figures. RNA levels of *IGF2* were also significantly increased after H_2_O_2_ exposure in both PPC1 (3-fold) and 9E6/E7 (1.5-fold), as shown in Figure S1. Therefore, oxidative stress induces biallelic *IGF2* expression in multiple cell lines, and the regulatory mechanisms underlying this event was examined in subsequent experiments.

### CTCF expression and binding to the H19 Imprint Control Region (H19-ICR) is reduced

**Figure 2:**
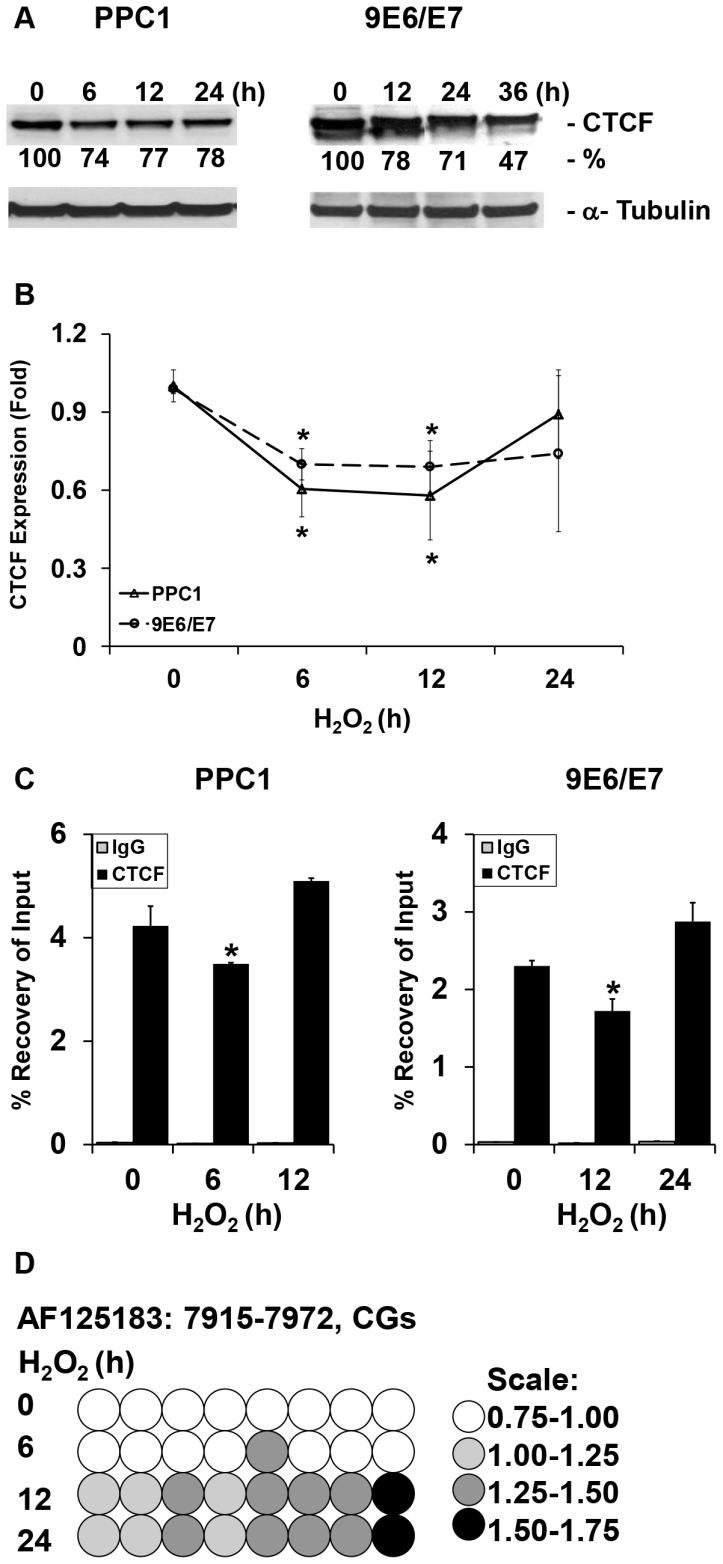
Oxidative stress results in CTCF reduction, loss of CTCF binding to and DNA hypermethylation across the H19-ICR region. (A) Oxidative stress represses CTCF protein expression in prostate cells. Whole lysates were extracted from treated cells (PPC1, left panel; 9E6/E7, right panel) and analyzed by Western blot analysis using an anti-CTCF antibody and tubulin as loading control. The blots were quantified using ImageJ and are shown as percentage of control. The CTCF decrease was statistically significant when calculated from three independent Western blots. (B) Oxidative stress decreases mRNA expression of *CTCF* in prostate cells. The mRNA levels of *CTCF* were measured using RT-qPCR. Values are expressed as mean+/−S.D. of three independent experiments measured in duplicates. * *P*<0.05 (t-test). (C) ChIP to evaluate CFCF binding demonstrates H_2_O_2_ exposure reduces recovery of CTCF at the H19-ICR. Values are expressed as mean+/−S.D * *P*<0.05 (t-test) and compared with IgG controls. (D) Oxidative stress induces progressive hypermethylation of the H19-ICR in PPC1 cells. Density of the circles represents fold change in methylation compared to the no treatment control. The ICR region is ∼2 kb upstream of H19 transcription start site and encompasses all CTCF binding site 6.

CTCF is a widely expressed protein that has been linked to epigenetic regulation [17] and demonstrates decreased expression during stress-induced apoptosis [13]. To determine if oxidative stress-induced cellular responses modulate CTCF, CTCF levels in PPC1 and 9E6/E7 cells were examined following exposure to H_2_O_2_. CTCF protein (Fig. 2A) and mRNA expression (Fig. 2B) were reproducibly decreased in these experiments.

The presence of CTCF on the human H19-ICR acts as an insulator to block enhancers from binding to and driving biallelic IGF2 expression [18]. To test whether CTCF binding is altered by oxidative stress, we employed chromatin immunoprecipitation (ChIP) using antibodies against CTCF. Cross-linked DNA was subjected to quantitative PCR analysis to assess the binding of CTCF to the H19-ICR. CTCF binding reproducibly decreases after exposure to H_2_O_2_ (Fig. 2C) in both cell lines.

### DNA methylation of the H19-ICR occurs with oxidative stress

Secondary DNA methylation may result after persistent CTCF loss and prevents further binding of the insulator protein [18]. We tested whether H_2_O_2_ alters DNA methylation across several CTCF binding sites within the human H19-ICR using quantitative pyrosequencing. We found that H_2_O_2_ exposure results in an accumulation of DNA methylation within the H19-ICR region in cells over time (Fig. 2D). This increased methylation was most noticeable across the 3′ end of the sequence that corresponds to CTCF binding site 6 in the human, a critical region in controlling allelic silencing. Methylation of the IGF2 promoter was not altered (data not shown).

### IκBα super-repressor inhibits CTCF downregulation and IGF2 LOI induced by oxidative stress

**Figure 3:**
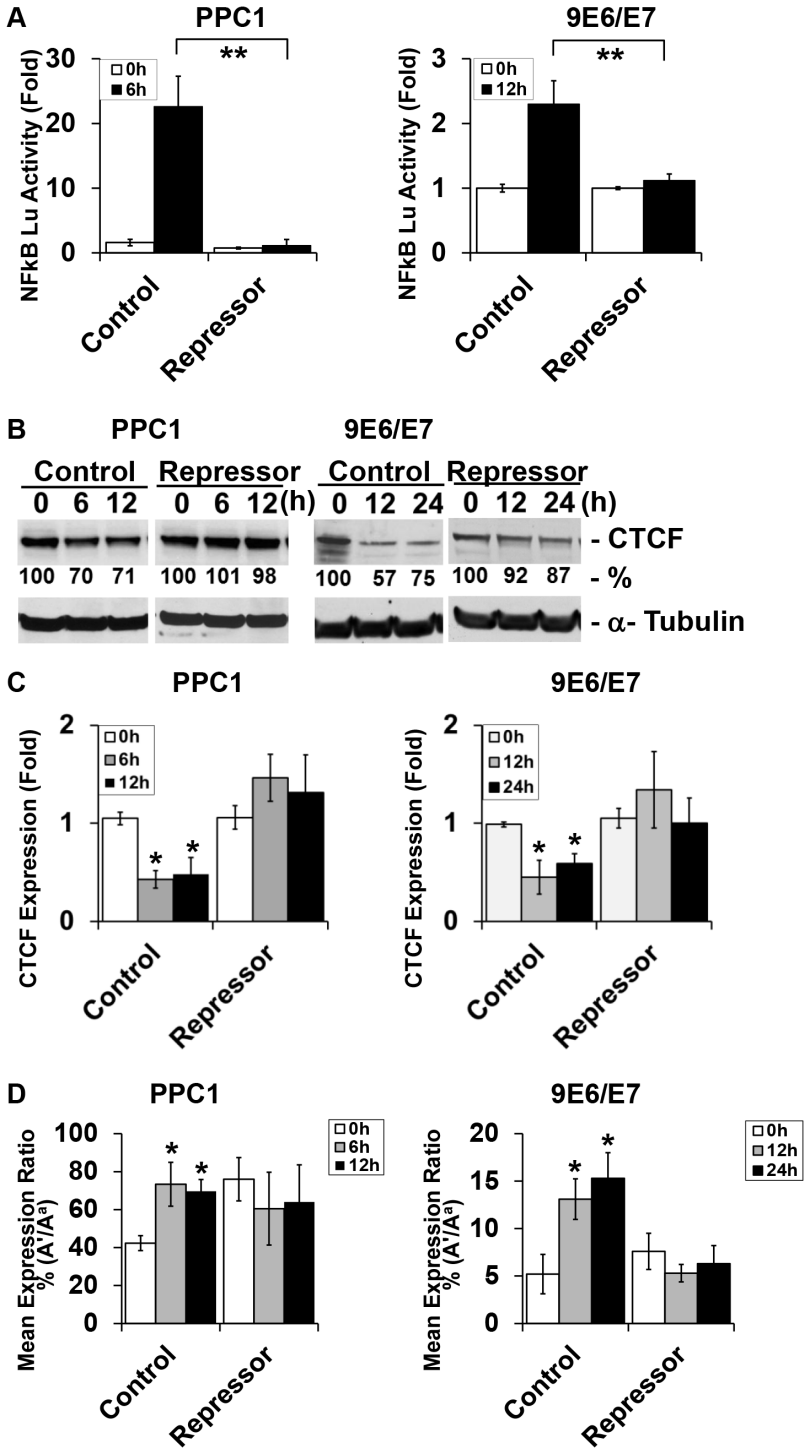
An IκBα super-repressor blocks NF-κB activity and *IGF2* LOI. (A) IκBα super repressor blocks oxidative stress-induced NF-κB activity. An IκBα super-repressor was utilized that contains mutant IκBα resistant to phosphorylation and degradation, thus blocking canonical NF-κB activation. Stable integration of this super-repressor or empty vector control was performed in PPC1 and 9E6/E7. These cells were then transduced with an NF-κB reporter for 48 hr and then treated with H_2_O_2_ for 6 hr. Values are expressed as mean+/−S.D. of three independent experiments. ** *P*<0.01 (t-test). (B) IκBα super-repressor inhibits oxidative stress-induced decreases in CTCF protein expression. Western blot of protein lysates were analyzed and quantitated. (C) IκBα super-repressor inhibits oxidative stress-induced decreases in CTCF mRNA expression. The mRNA levels of *CTCF* were measured using RT-qPCR in the cells. (D) IκBα super-repressor blocks loss of *IGF2* imprinting. FLuPE analysis of RNA was performed to evaluate allele-specific expression.

It was then determined whether *IGF2* LOI induced by H_2_O_2_ is dependent on the activation of NF-κB signaling. To specifically inactivate canonical NF-κB signaling, a retroviral construct harboring super-repressor IκBα mutant (or control) was stably transfected into PPC1 and 9E6/E7 cells. The stable cell lines were then transiently transduced with the NF-κB-dependent luciferase reporter gene for 48 hr and subsequently treated with H_2_O_2_. NF-κB reporter activity was induced in PPC1 (14 fold) and 9E6/E7 (2.3 fold) (Fig. 3A) in empty vector control lines. NFκB activity was not significantly altered in the super-repressor stable cells indicating effective blocking of NF-κB.

CTCF expression and *IGF2* imprinting were quantitated in control and super-repressor cell models. The downregulation of CTCF protein (Fig. 3B) and mRNA (Fig. 3C) by H_2_O_2_ was effectively blocked in the super-repressor cells when compared to controls. The super-repressor also prevented *IGF2* LOI induced by H_2_O_2_ (Fig. 3D). Therefore, inhibition of NF-κB activity with the super-repressor IκBα reversed the effect of oxidative stress on the suppression of CTCF expression and *IGF2* LOI in human prostate cells.

### Activation of NF-κB subtypes

**Figure 4:**
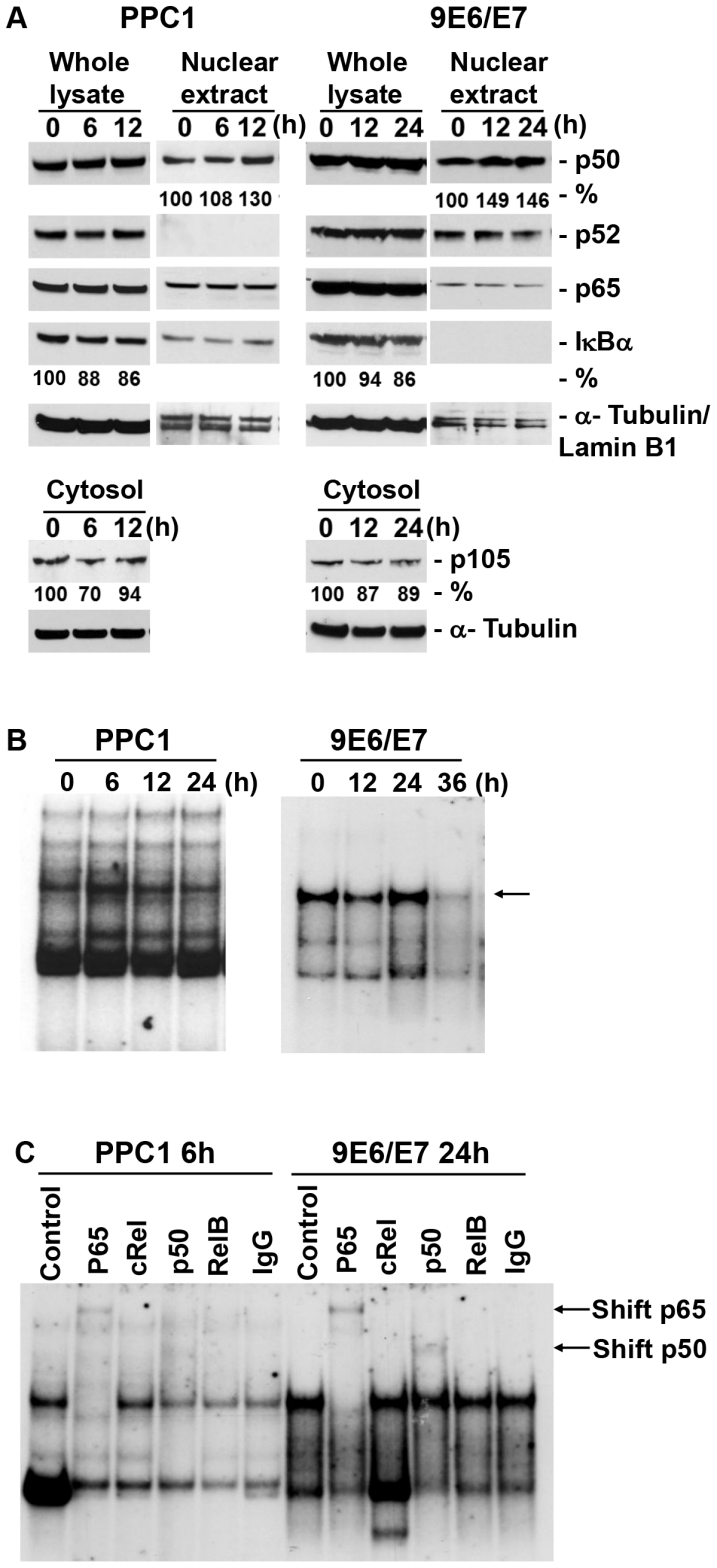
Oxidative stress induces nuclear translocation and subtype-specific activation of NF-κB in prostate cells. PPC1 and 9E6/E7 cells were treated with H_2_O_2_ for the times indicated, then used for extraction of either whole cell lysates or cytosol and nuclear fractions. (A) Induction of p50 and repression of IκBα with oxidative stress. NF-κB member protein levels were analyzed by Western blot using antibodies to specific NF-κB subtypes. Tubulin and LaminB1 were utilized as loading controls. (B) NF-κB DNA binding increases after exposure to H_2_O_2_. NF-κB DNA binding activity was analyzed by EMSA. NF-κB binding in whole cell extracts prepared from indicated cell samples was analyzed using a Igκ-κB probe. The location of NF-κB is indicated by the arrow. Free probe is not shown. (C) Supershift analysis of DNA-binding activity of NF-κB indicates p50 and p65 binding (arrows). For supershift analysis, 1 µg of antibodies against p65, c-Rel, p50 and RelB subunits, or control IgG, were included in the binding reaction prior to EMSA.

NF-κB signals through canonical and non-canonical pathways. To further interrogate these mechanisms, the accumulation of NF-κB protein subtypes and IκBα level were evaluated. Increased nuclear accumulation of p50 (30–49%) and decreased cytosolic p105 (13–30%) were found in both cell lines after H_2_O_2_ exposure (Fig. 4A). This correlated with a reduction of IκBα in whole cell lysates of both cell lines. There was minimal expression of cRel, therefore this protein was not examined further. Noncanonical pathway p52 proteins were not altered.

To independently assess the activation of NF-κB by H_2_O_2_, NF-κB DNA binding activity was analyzed by electrophoretic mobility shift assay (EMSA) (Fig. 4B). H_2_O_2_ induced the activation of NF-κB in PPC1 at 6 hr and in 9E6/E7 at 24 hr. The above time points were selected to further identify the specific NF-κB members activated by H_2_O_2_ using supershift analysis (Fig. 4C). Supershifted bands (indicated) compared to IgG controls indicated that H_2_O_2_ induced an increase in the DNA-binding activities of p50 and p65 in both cell lines. These results implicate the binding and involvement of canonical NF-κB pathway proteins in the cellular response to oxidative stress.

### Identification and occupancy of NF-κB binding sites within the CTCF promoter

**Figure 5:**
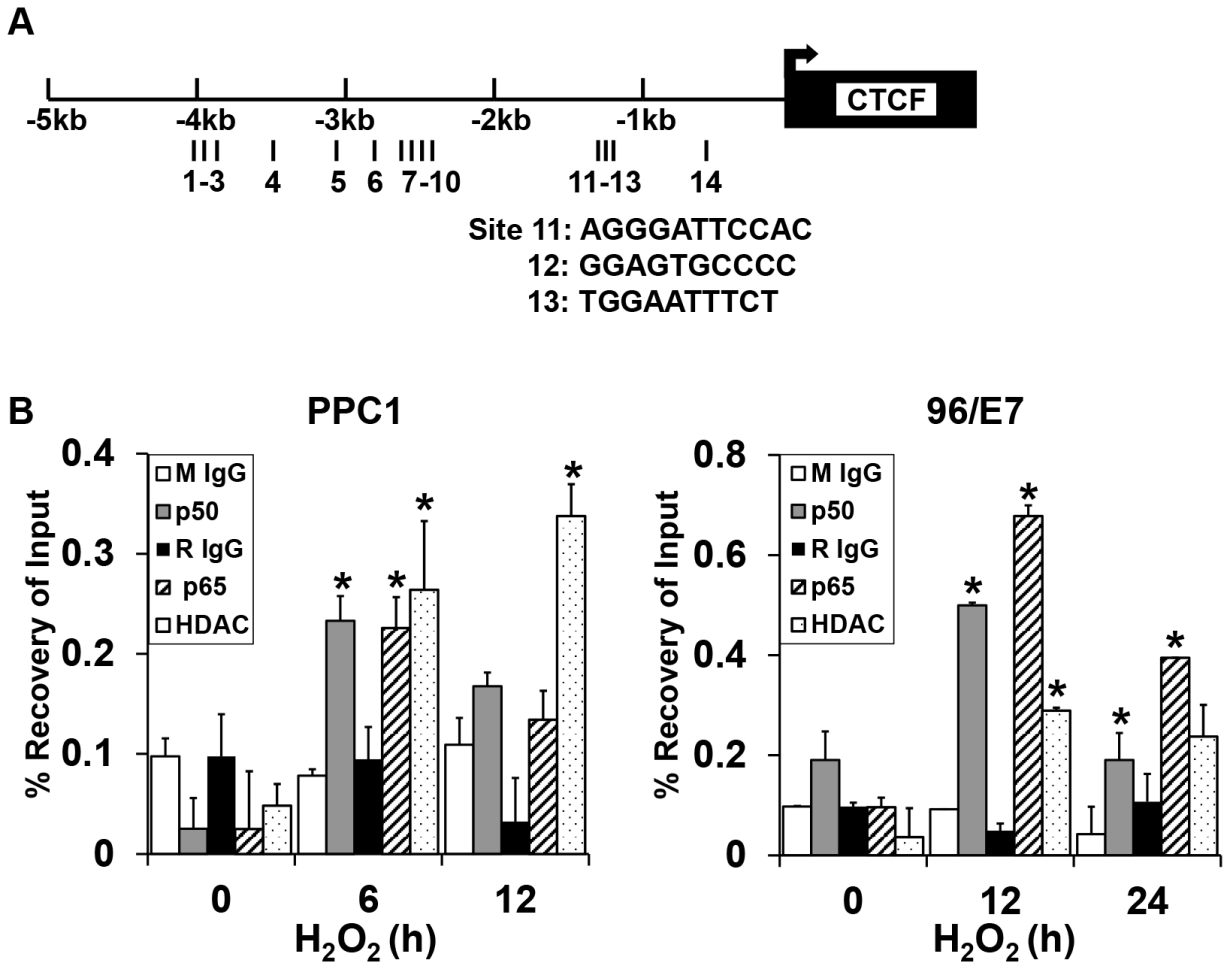
Increased binding of NF-κB subtypes p50 and p65 to the human CTCF promoter. (A) Schema showing the location of 14 putative NF-κB binding sites in CTCF promoter region relative to the CTCF transcription start site. These were identified using the JASPA database. Binding of NF-κB was assessed across all of these putative NF-κB binding sites. (B) ChIP-based qPCR for NF-κB binding demonstrates H_2_O_2_ exposure induces significant recovery of both p50 and p65 at CTCF region 11–13. Other binding sites evaluated did not show alterations in binding. HDAC binding also increases consistent with repression of the target gene. Values are expressed as mean+/−S.D * *P*<0.05 (t-test), compared with IgG controls.

To further delineate the NF-κB regulation of CTCF gene transcription under oxidative stress, the presence of potential NF-κB binding sites in the CTCF promoter region was determined using the JASPA database. We identified 14 such binding sites (Fig. 5A and Table S1). To test whether NF-κB binds to the CTCF promoter region, we employed chromatin immunoprecipitation (ChIP) using antibodies against NF-κB proteins p50 and p65 that were found to be activated by H_2_O_2_ above (Fig. 4C). The cross-linked DNA that was precipitated by either p50 or p65 antibody was subjected to quantitative PCR analysis to screen for binding at all the putative κB sites. We found that both p65 and p50 were consistently recruited to the CTCF promoter region containing κB sites (11–13) in both cell lines in response to H_2_O_2_ treatment at time points consistent with the gene repression (Fig. 5B). Other sites were interrogated and served as negative controls (Figure S2).

NF-κB differentially regulates target gene expression via the recruitment of transcriptional co-regulators [19], [20]. The recruitment of the co-activator CBP and co-repressor HDAC1 to the CTCF promoter in association with NF-κB recruitment was tested. H_2_O_2_ exposure enhanced binding of HDAC1 to the CTCF promoter in repeated experiments, consistent with the down-regulation of CTCF (Fig. 5B). CBP binding was not altered (data was not shown). These data support the placement of CTCF gene repression downstream of the stress-induced NF-κB pathway and implicate the canonical pathway.

### NF-κB activation induces IGF2 LOI in the mouse prostate

A mouse model was then utilized to determine whether NF-κB activation alone leads to *IGF2* LOI, as well as testing this regulatory pathway *in vivo*. The mice employed contain a mutant IκBα which results in the constitutive activation of NF-κB in many tissues including the prostate [21]. C57BL/6 IκBα+/− females with constitutive NF-κB activation were crossed with B6 (*Mus Cast* H19-p57) males containing an *IGF2* polymorphism (A/G). Prostate tissues were harvested at 1 mo. Histopathology demonstrated no overt changes in glandular structure of prostate tissues.

**Figure 6:**
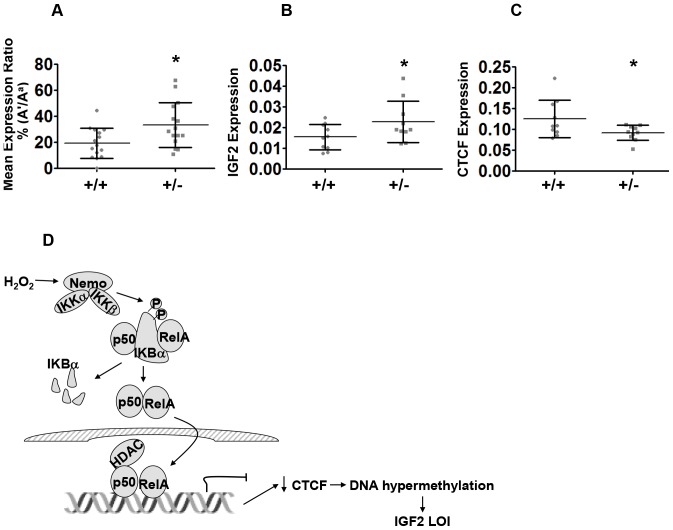
NF-κB activation in mice with an IκBα mutation induces *IGF2* LOI in the mouse prostate. (A) Prostate tissues were dissected from informative male IκBα+/+ and IκBα+/− mice (1 mo) and RNA obtained. Maternal and paternal allelic expression was measured using FluPE. The ratio of the inactive allele (Ai) to active allele (Aa) was calculated for each age cohort (n = 15, *, *P*<0.05). Increased LOI was demonstrated in the 1 mo mouse DLPs. (B) *IGF2* expression increases in 1-month IκBα+/− mouse DLPs. RT-qPCR was used to measure *IGF2* expression levels in the mouse DLPs of the 1 mo-old cohorts. Values are expressed as mean+/−S.D (n = 10, * *P*<0.05). (C) CTCF expression decreases in 1-month IκBα+/− mouse DLPs. RT-qPCR was used to measure CTCF expression levels in the mouse DLPs of the 1 mo-old cohorts. Values are expressed as mean+/−S.D (n = 10, * *P*<0.05). (D) Schematic of NF-κB and CTCF pathway. Hydrogen peroxide stimulates the activation of IKKβ, resulting in the phosphorylation of IκBα at Ser 32 and Ser 36. This leads to its degradation with release of the NF-κB (p65/p50 heterodimer) and subsequent translocation to the nucleus. NF-κB binds to the promoter region of CTCF, with the corepressor HDAC1 and reduces transcription of the CTCF gene. Decreased expression of CTCF results in DNA hypermethylation and *IGF2* LOI.

RNA was isolated, *IGF2* imprinting was quantitated using FluPE and RNA expression was examined by qRT-PCR. We previously observed a lobe-specific LOI involving the mouse DLP, a homologue for the human peripheral prostate, during aging [6]. DLP tissues from 1 mo IκBα+/− mice demonstrate reactivation of the silenced allele when compared to wild type (WT) counterparts (Fig. 6A). The IκBα+/− animals containing activated NF-κB also express increased *IGF2* (Fig. 6B). No significant relaxation in *IGF2* imprinting was observed in the ventral prostate (data not shown). CTCF mRNA levels decreased in 1 mo IκBα+/− mice compared to the wild type mice (Fig. 6C). These *in vivo* results support a direct role for NF-κB in modulating CTCF levels and imprinting.

## Discussion

There is mounting evidence that oxidative stress may impact not only the genome, but epigenetic elements as well. The regulatory factors linking inflammation to epigenetics have not been well defined. One candidate is CTCF, a regulatory protein with 11 highly conserved zinc finger domains that plays an important role in transcription, but recent data suggest a role in modulating the epigenome. The presence of CTCF prevents DNA methylation of CG-enriched regions *in vitro* [8]. Our laboratory has previously found that during aging, CTCF is decreased in the prostate associated with a loss of the normal imprint at *IGF2* [6]. The *IGF2*-H19 locus is a well-characterized epigenetic target with important implications in cancer development. In the present study, we establish a novel mechanistic link between oxidative stress and *IGF2* imprinting through NF-κB-mediated repression of CTCF expression and binding to the H19-ICR region. This NF-κB/CTCF response occurs in both human prostate cells *in vitro* and in prostate tissues from mice that have higher basal NF-κB activity. This observation mechanistically links the regulation of imprinting to oxidative stress, an observation important in aging and cancer.

Imprinting of the *IGF2* gene is driven primarily by the binding of the insulator CTCF to the H19 ICR in both the human and the mouse [7], [22], [23]. Exposure to H_2_O_2_ results in *IGF2* LOI in both cell lines tested. This LOI was calculated as a percentage of the expressed allele and was likely underrepresented given the multiple 11p15 copies seen in these cell lines. LOI was confirmed in a mouse prostate containing multiple cell types (Fig. 6). This biallelic expression was associated with reproducible CTCF loss of binding and expression consistent with known models. The regulation of CTCF is complex and poorly studied. However, CTCF downregulation has been observed after cell exposure to UV radiation [24].

CTCF is a dynamic protein whose loss of binding leads to hypermethylation of CpG-enriched regions [8]. An increase in DNA methylation across the H19-ICR consistent with this previous observation was observed (Fig. 2D). Regional hypermethylation at this CTCF binding site is in contrast to previous observations that oxidative stress globally decreases methylation in mouse models deficient in CuZnSOD [25], a result of DNA adducts inhibiting DNA methyltransferase [26]. The increase in methylation at the H19-ICR region occurred after CTCF reduction and binding, suggesting that these methylation changes are due to decreased CTCF occupancy and not directly caused by oxidative stress. The hypermethylation found suggests other higher order epigenetic changes, including histone modifications, may also be altered by oxidative stress and would be a target for future study.

The activation of NF-κB occurs through distinct canonical and noncanonical pathways. The canonical pathway involves the activation of the NFκB subunits p50 and p65/RelA and is most consistent with our expression and binding data. Other research supports a noncanonical pathway promoting activation of the redox-sensitive NIK/IKK pathway [27], [28]. The current data did not observe the activation of p52 or RelB after exposing the cells to H_2_O_2_. H_2_O_2_ directly induces phosphorylation of IκBα at Tyr42, resulting in its degradation and dissociation from p50/p65, which induces an atypical IKK-independent NF-κB activation [29], [30]. To further interrogate this pathway, we used a super-repressor containing mutations at position S32 and S36 of IκBα that prevents IKKβ-mediated phosphorylation. This abolished the effects of H_2_O_2_ on NF-κB activation and CTCF downregulation (Fig 3) providing further evidence for canonical activation.

The activation of NF-κB results in the induction or suppression of downstream genes depending on the presence and binding of different dimers [31]. With low dose H_2_O_2_ exposure, we observed the induction and binding of a p65/p50 heterodimer to the CTCF promoter. The presence of a NF-κB binding site on the CTCF promoter has been previously recognized [24]. This study with EGF induction and UV light involved both p65/p50 heterodimers, as well as p50 homodimer formation. While p65/p50 heterodimers generally activate target gene transcription [32], transcriptional outcomes are subject to the regulation of a dynamic balance between coactivators and corepressors [19], [33]. Our ChIP data indicated that the corepressor HDAC1 was recruited to the CTCF gene promoter in association with p50 and p65 resulting in decreased CTCF expression. This occurred at a specific region in the CTCF promoter (sites 11–13). Other sites failed to demonstrate significant p65/p50 binding and were used as controls (Figure S2).

To mechanistically verify that the downregulation of CTCF was mediated through NF-κB signaling, we utilized two approaches. First, we introduced an IκBα super-repressor in which mutations at IκBα phosphorylation sites render it unresponsive to canonical upstream inducers. This super-repressor robustly blocked NF-κB activity and CTCF downregulation. Secondly, we employed IκBα+/− mice which directly induced higher basal NF-κB activity. These IκBα+/− animals have increased activation of NF-κB in the prostate [21]. Using a polymorphism to identify different alleles, we demonstrate that the activation of NF-κB alone leads to increased *IGF2* LOI in mouse prostate and decreased CTCF expression when compared to WT (Fig 6). These IκBα+/− mice also demonstrate increased prostate cancer risk with aging when utilized in genetic models [34]. Through the use of these two approaches, the important role of the NF-κB/CTCF pathway in controlling *IGF2* imprinting was confirmed. We do not, however, discount other minor effects that H_2_O_2_ might have on *IGF2* biallelic expression including altering other transcription factors.

The significance of the current study lies in the elucidation of a mechanism for oxidative stress to promote altered imprinting through canonical NF-κB signaling. Inflammation plays an important role in the development of age-related cancers, but mechanistic data linking inflammation to epigenetic alterations has been lacking. It is anticipated that antagonists of inflammation that inhibit NF-κB, including the spice curcumin [35] and diterpenes found in coffee [36], would modulate imprinting. Our study also suggests a pivotal role for CTCF in modulating not only imprinting, but potentially regional hypermethylation. CTCF levels have been found to decrease with aging and cancer [6]. Finally, these observations may help explain the altered epigenetic landscape seen with aging that underlies the increased risk of cancer.

## Materials and Methods

### Cell lines and treatment

The PPC-1 prostate cancer cell line was obtained from the ATCC, and E6/E7 is one of a series of nontumorigenic, immortalized lines derived from human prostate epithelial cells [16]. Hydrogen peroxide (Sigma, St. Louis) was used to induce oxidative stress. Dose and time-dependent experiments were performed on both cell lines to maximize induction of NF-κB activity with minimal apoptosis. At each time point, cells were trypan blue-stained for viability and apoptosis was detected with Annexin V. Final doses were chosen at 800 µM for PPC1 and 1600 µM for 9E6/E7 cells.

### NF-κB activity

A Lenti-based NF-κB-responsive firefly luciferase reporter (SABiosciences, Frederick) was used to monitor the activity of NF-κB-regulated signal transduction pathways as instructions from the company. Briefly, lentivirus containing the NF-κB reporter or negative control reporter was applied to target cells at an MOI 25. Culture media were changed after 20 hours and puromycin was added at 48 hr for selection of stable cell lines. In order to determine the appropriate amount of puromycin for selection, a titration was performed in both cell lines (PPC1, 1 µg/ml; 9E6/E7, 0.5 µg/ml). The stable cells were expanded and then plated into 24-well plate and treated with H_2_O_2_ at indicated time points and then rinsed with PBS and lysed in 100 µl of Cell Culture Lysis Reagent (Promega, WI). The Luciferase Assay then was used on 10 µl of cell lysate in 100 µl of the Luciferase Assay reagent (Promega) and measured with a luminometer-Monolight 2010. Every treatment was done as triplicates and each experiment performed three times separately.

### NF-κB inhibition

A pBabe-Puro-IκBα-mut (super-repressor, Addgene, MA) retroviral construct was used to inhibit NF-κB activity. The IκBα super repressor harbors two amino acid substitutions (S32A/S36A) which renders this mutant IκBα resistant to phosphorylation and degradation, thus blocking canonical NF-κB activation [37]. The retrovirus was packaged using a Retrovirus Kit Ampho (TAKARA Bio Inc. Otsu, Shiga, Japan) in 293FT cells (ATCC) per manufacturer's instructions. The recombinant retrovirus particles were tittered and used to infect (5×10^5^/100-mm dish) cells with 10^5^ infectious viral units, total final volume was 5 ml. Selection was performed for 2 weeks and then split into either 24-well plate for the NF-κB activity assay or P-100 plate for detection of gene and protein expression.

### Imprinting and expression measurement

RNA was isolated from the cells or mouse prostate tissues using Rneasy Kit (Qiagen) with the addition of Dnase I to minimize DNA contamination. Imprinting was performed using a FluPE assay as previously described [6]. For human *IGF2*, a single nucleotide polymorphism identified on *IGF2* exon 7 (G/C) was used to identify individual alleles. *IGF2* imprinting was examined on Exon 6 (A/G) in mouse prostate tissues. Differences were determined by calculating the ratio of their respective spectral intensities [repressed allele (Ai)/active allele (Aa)]. Quantitative PCR was performed using an iCycler (Bio-Rad) and SYBR Green PCR master mix (Applied Biosystems) to measure gene expression, primers are available on request. Western blot was performed to detect protein expression using antibodies for CTCF (Cat #07-729, Millipore), NF-κB p50 (4D1, Santa Cruz), NF-κB p65 (c-20, Santa Cruz), NF-κB p100/52 (18D10, cell signaling), IKKα/β (sc-7607, Santa Cruz) and IκBα (c-21, Santa Cruz) and α-Tubulin (DM1A, EMD).

### EMSA and supershift assay

Supershift assays were used to identify which subunit of NF-κB is activated by H_2_O_2_. Briefly, electrophoretic mobility shift assay (EMSA) was done according to O'Conner *et al* [38]. Treated and untreated cells were harvested, washed twice with 1×PBS and whole cell extracts were obtained by lysis in Totex buffer containing HALT protease inhibitors (Pierce, IL). The concentration of proteins was estimated by a Bradford assay. Ten µg of extracts (∼2 µL) were incubated in 9 µL binding buffer containing 1 pg of poly(dI-dC) (Pharmacia) for 20 min on ice and then 1 µL of ^32^P-labeled double-stranded oligonucleotides containing the κB site (underlined) from the Igκ gene (5′-TCAACAGAGGGGACTTTCCGAGGCC-3′) was added and incubated for additional 20 min at room temperature. The NF-κB bound and free κB probes were resolved by electrophoresis via 4% native PAGE gel. The dried gels were exposed to X-ray film and Phosphoimage cassette for quantitation by ImageQuant analysis. For supershift assays, 1 µg of IgG antibodies specific to members of the NF-κB proteins (p65, c-20; cRel, 5071[39]; p50, NLS; RelB, c-19, Santa Cruz) were added to nuclear extracts for 20 min on ice prior to addition of radiolabeled probe.

### Chromatin Immunoprecipitation (ChIP)

To assess NF-κB binding to the CTCF promoter region we analyzed the 5000-bp DNA sequence upstream from the 1^st^ exon of CTCF gene with the JASPAR database, and found 14 putative binding sites for NF-κB (Fig. 5A and Table S1a). The ChIP assay was performed as previously described [40] to detect NF-κB interaction with the CTCF promoter region. Briefly, protein was crosslinked to chromatin DNA in 1% formaldehyde and immunoprecipitated using the following antibodies: p50, p65, CBP, HDAC1 (Santa Cruz), rabbit or mouse control IgG (Millipore, MA). After immunoprecipitation, the purified DNA was amplified by quantitative PCR with the primers listed (Table S1), which are specific to the NF-κB binding sites. β-actin was used as a negative control for NF-κB target gene. Quantitative real-time PCR data are presented by setting the control IgG-precipitated samples as 1, and input was used for normalization. The average and S.D. values were calculated and plotted by the Microsoft Excel. CTCF binding to the H19-ICR (imprint control region) was performed as same as above with the exception that we used a CTCF antibody (Millipore, MA) for immunoprecipitation and different primers (Table S1b) flanking human CTCF binding site 6 within H19-ICR for quantitative PCR.

### Methylation analyses

To detect whether H_2_O_2_ will affect DNA methylation, we evaluated DNA methylation status in two regions. The H19-ICR, 2 kb upstream of the H19 start site (GenBank accession no. AF125183) was assessed, and the *IGF2* DMR0 found in the promoter [41]. DNA isolation, bisulfite modification and quantitative bisulfite pyrosequencing were done as described previously [42]. The primer sequences for the H19-ICR are listed in Table S1c, and for DMR0 are as Murrell *et al* reported [41].

### Mice with continuous NF-κB activation

IκBα deficient mice were kindly provided by Drs. Kerr and Yull (Vanderbilt University). As described [43], the IκBα locus was disrupted by homologous recombination in ES cells using two targeting vectors that replaced the promoter and first exon of the IκBα gene with a PGK-Neo cassette. This strategy was designed to disrupt IκBα transcription and translation. IκBα−/− pups stop gaining weight and typically die within 9 days after birth, so only IκBα+/− mice were utilized. NF-κB signaling is continuously overactivated in the prostate of the IκBα+/− mouse. B6 (Cast H19-p57) mice were obtained from Dr. Shirley Tilghman (Princeton University, Princeton, NJ) [6]. Male mice homozygous for Mus castaneus alleles (H19-p57) were bred with female C57BL/6 IκBα+/−; the offspring contain a polymorphism (A/G) within *IGF2* exon 6. Male mice from each litter were entered randomly into different experimental time points. Fifteen animals per time point were euthanized at intervals (every 4 weeks) beginning at age 1 month. Tissues were microdissected, including the coagulating glands, dorsolateral prostate (DLP), and ventral prostate (VP), and placed in RNA-later for RNA isolation or in 10% formalin for histopathology analysis. This study was approved by Institutional Animal Care and Use Committee at University of Wisconsin-Madison.

## Supporting Information

File S1 **Contains Table S1 and Figures S1 and S2. Figure S1.** Oxidative stress induces increased expression of *IGF2* in prostate cells. The mRNA levels of *IGF2* were measured using RT-qPCR in the cells. Values are expressed as mean+/−S.D. of three independent experiments measured in duplicates. * *P*<0.05 (t-test). Figure S2. Identifying binding of the NF-κB protein to the human CTCF promoter. ChIP-based qPCR for NF-κB binding demonstrates H_2_O_2_ exposure did not affect recovery of both p50 and p65 at CTCF region 7–10. Values are expressed as mean+/−S.D * *P*<0.05 (t-test), compared with IgG controls.
(PDF)
