# Supplementary material for: A Novel Pathway Links Oxidative Stress to Loss of Insulin Growth Factor-2 (IGF2) Imprinting through NF-κB Activation
Source: PLoS One. 2014 Feb 18;9(2):e88052. doi: 10.1371/journal.pone.0088052 (PMC3928145; doi:10.1371/journal.pone.0088052)

Table S1.

a. Sequences of NFkB binding sites in CTCF promoter region and primers for ChIP assay

site 1: AGGCTTTTTTCCC
No primers successfully designed

site 2: TGTAATCCCA **, 3**: GGCAAAACCCC
Primer: F: TTTTTC CCTCTA AATCCACTGC
R: TTT AGTGG AGACGGGGTTTT

site 4: GGAAGGCCAA
Primer: F: GACAGGTGTGGTGGCTTACA
R: TGGAGATGAGGTCTCACTGTGT

site 5: TGGGATTACA
Primer: F: CCTGGGCTCAAGTAATCCAC
R: CCTGGGTGATGAAGCAAGAC

site 6: GGTAATTTTC
Primer: F: GGCTGAGGAGAATGCAAAAT
R: GCCTCTTATGTACACATTAGGACTCA

site 7: TGTAATCCCA ; **8:** TGGGAGTTCA
9: GGAGAAACCCCA; **10:** TGTAATCCCA
Primer: F: CGGTGAGTCGCTCCTGTAAT
R: TCAGCTCACCACAACCTCTG

site 11: AGGGATTCCAC; **12:** GGAGTGCCCC
13: TGGAATTTCT
Primer: F: AGCTGATGCCTCCTACCTGA
R: TTGGCTTGGAGGAAAGAGTG

site 14: CTGGTTTTCC
Primer: F: AAGCGATTGCTATGAACGTG
R: GGTGCGAGAGGAGAGTGG

b. Primer sequences for detecting CTCF binding in H19-ICR

H19CTCF
F: GAGGCTTCTCCTTCGGTCTCA
R: GCCACTTCCGATTCCACAAC

c. Primer sequences for checking the H19-ICR methylation by Pyrosequencing

Region 1:
F: GGATGGTAAGGAATTGGTTGTAGTT
R: ACTCCCATAAATATCCTATTCCC
Sequencing: TAAATATCCTATTCCCAAAT

Region 2:
F: GGGGGTTTTTGTATAGTATATGGGTA
R: ACTCCCATAAATATCCTATTCCC
Sequencing: GGGTATTTTTGGAGGTT

Figure S1.

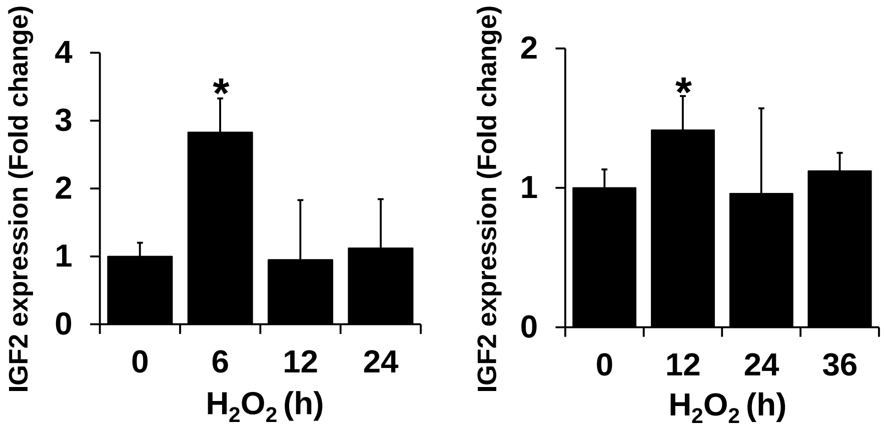

Figure S2.

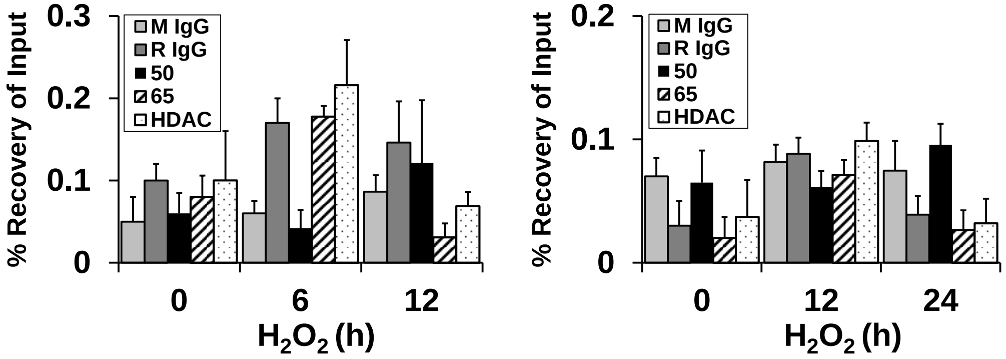

Supplement: File S1 — Contains Table S1 and Figures S1 and S2. Figure S1. Oxidative stress induces increased expression of IGF2 in prostate cells. The mRNA levels of IGF2 were measured using RT-qPCR in the cells. Values are expressed as mean+/−S.D. of three independent experiments measured in duplicates. * P<0.05 (t-test). Figure S2. Identifying binding of the NF-κB protein to the human CTCF promoter. ChIP-based qPCR for NF-κB binding demonstrates H2O2 exposure did not affect recovery of both p50 and p65 at CTCF region 7–10. Values are expressed as mean+/−S.D * P<0.05 (t-test), compared with IgG controls. (PDF) [file pone.0088052.s001.pdf]
